# Autophagy-Enhancing Properties of *Hedyotis diffusa* Extracts in HaCaT Keratinocytes: Potential as an Anti-Photoaging Cosmetic Ingredient

**DOI:** 10.3390/molecules30020261

**Published:** 2025-01-10

**Authors:** Qiwen Zheng, Xiangji Jin, Trang Thi Minh Nguyen, Eun-Ji Yi, Se-Jig Park, Gyeong-Seon Yi, Su-Jin Yang, Tae-Hoo Yi

**Affiliations:** 1 Graduate School of Biotechnology, Kyung Hee University, Yongin-si 17104, Republic of Korea; zhengqiwen@khu.ac.kr (Q.Z.); trangnguyen@khu.ac.kr (T.T.M.N.); 0201@khu.ac.kr (E.-J.Y.); tpwlt@khu.ac.kr (S.-J.P.); stella@khu.ac.kr (S.-J.Y.); 2 Department of Dermatology, School of Medicine, Graduate School, Kyung Hee University, Seoul 02447, Republic of Korea; hyanghe112@khu.ac.kr; 3 Department of Biopharmaceutical Biotechnology, Graduate School, Kyung Hee University, Yongin-si 17104, Republic of Korea; ks010924@khu.ac.kr

**Keywords:** autophagy-enhancing, *Hedyotis diffusa*, anti-photoaging, HaCaT keratinocytes

## Abstract

The decline in autophagy disrupts homeostasis in skin cells, leading to oxidative stress, energy deficiency, and inflammation—all key contributors to skin photoaging. Consequently, activating autophagy has become a focal strategy for delaying skin photoaging. Natural plants are rich in functional molecules and widely used in the development of anti-photoaging cosmetics. *Hedyotis diffusa* (HD), as a medicinal plant, is renowned for its anti-inflammatory and anticancer properties; however, its effects on skin photoaging remain unclear. This study investigates HD’s potential to counteract skin photoaging by restoring mitochondrial autophagy in keratinocytes. We used HPLC to detect the main chemical components in HD and, using a UVB-induced photoaging model in HaCaT keratinocytes, examined the effects of HD on reactive oxygen species (ROS) levels, Ca^2+^ concentration, mitochondrial membrane potential (MMP), apoptosis, and the cell cycle. Cellular respiration was further evaluated with the Seahorse XFp Analyzer, and RT-PCR and Western blotting were used to analyze the impact of HD on mitochondrial autophagy-related gene expression and signaling pathways. Our findings indicate that HD promotes autophagy by modulating the PI3K/AKT/mTOR and PINK/PARK2 pathways, which stabilizes mitochondrial quality, maintains MMP and Ca^2+^ balance, and reduces cytochrome c release. These effects relieve cell cycle arrest and prevent apoptosis associated with an increased BAX/BCL-2 ratio. Thus, HD holds promise as an effective anti-photoaging ingredient with potential applications in the development of cosmetic products.

## 1. Introduction

Chronic UVB exposure accelerates skin photoaging, which differs from age-related aging in that it can be reversed with appropriate interventions [1]. In the epidermis, keratinocytes are the primary cell type directly impacted by the harmful effects of UVB radiation. UVB primarily acts through chromophores, such as DNA, which absorb UVB and trigger a cascade of biological effects. This absorption leads to the generation of reactive oxygen species (ROS) as secondary byproducts, further contributing to cellular damage [2]. Notably, the UVB-induced HaCaT keratinocytes model effectively simulates the early pathological processes of skin photoaging and is, therefore, widely used in skin photoaging research [3]. Upon UVB exposure, it typically initiates a series of intracellular stress responses, including oxidative stress, inflammation, and DNA damage [4,5]. These insults disrupt autophagy, leading to the accumulation of dysfunctional mitochondria. This triggers cell cycle arrest, increases apoptosis, and worsens skin photoaging while also raising cancer risk [6,7]. Consequently, restoring mitophagy function has emerged as a promising strategy for mitigating the progression of skin photoaging [8]. Furthermore, recent studies have highlighted the systemic effects of UV radiation, showing that it not only affects the skin but also impacts the brain and immune system [9].

Mitochondria, often referred to as the “powerhouses” of eukaryotic cells, play a pivotal role in maintaining cellular function and stability by regulating Ca^2+^ balance, producing metabolic intermediates, and controlling reactive oxygen species (ROS) production [10]. Efficient mitochondrial function depends on the dynamic regulation of mitophagy, a selective form of autophagy that removes damaged mitochondria to maintain cellular homeostasis, a process central to research on age-related neurodegenerative diseases [11]. Recently, the importance of mitophagy in skin photoaging has gained increasing attention [12]. Cavinato et al. demonstrated that NIX-dependent mitophagy alleviates skin photoaging by clearing damaged mitochondria [13]. Additionally, nicotinamide has been shown to restore mitochondrial integrity and improve mitophagy, exerting anti-photoaging effects [14]. The PI3K/AKT/mTOR pathway is an effective target in autophagy regulation. Meanwhile, isoliquiritigenin induces melanin degradation in human epidermal keratinocytes by promoting autophagy through the PI3K/AKT/mTOR pathway [15]. Although its role in UVB-induced skin photoaging has been less studied, it has been widely investigated in mitophagy regulation in cancer [16], neurodegenerative diseases [17], and atherosclerosis [18]. Based on this, we hypothesize that the PI3K/AKT/mTOR pathway may also play a key role in addressing mitochondrial autophagy dysfunction in photoaging.

Simultaneously, UVB radiation induces leakage in the mitochondrial electron transport chain, particularly in complexes I and III, leading to the production of superoxide anions and other ROS [19,20]. This results in the accumulation of damaged mitochondria within cells, becoming a primary trigger for the ROS surge following UVB exposure. In this process, nuclear factor erythroid 2-related factor 2 (Nrf2) serves as a crucial regulator by countering ROS-induced mitochondrial damage [21,22]. Specifically, Nrf2 facilitates mitophagy regulation by upregulating antioxidant enzymes such as heme oxygenase-1 (HO-1) and NAD(P)H quinone oxidoreductase-1 (NQO-1) [3]. For instance, studies have shown that urolithin A can alleviate UVA-induced photoaging in human dermal fibroblasts by activating Nrf2 and promoting mitophagy [23]. Additionally, other studies indicate that Nrf2 activation reduces UVB-induced ROS production through antioxidant mechanisms, thereby mitigating mitochondrial damage [24]. Thus, the auxiliary regulation of the Nrf2 signaling pathway supports the maintenance of mitochondrial homeostasis.

Medicinal plants have attracted increasing attention in cosmetic product development due to their natural origin and eco-friendly properties [25]. *Hedyotis diffusa* (HD), a traditional medicinal plant from the Rubiaceae family, is widely used to prevent and treat tumors, urinary system disorders, and gastrointestinal diseases [26]. Moreover, HD has demonstrated promising anti-aging effects in *Caenorhabditis elegans* models and exhibited therapeutic potential in Alzheimer’s disease [27,28]. It contains bioactive compounds such as quercetin and chlorogenic acid. Quercetin has been shown to regulate mitochondrial biogenesis, membrane potential, oxidative respiration, and ATP synthesis, thereby preventing mitochondrial damage [29]. Meanwhile, chlorogenic acid enhances cellular autophagy, mitigating oxidative stress-induced damage [30]. Despite these beneficial properties, research on the application of HD in skin photoaging has not yet been reported.

This study used a UVB-induced photoaging cell model to investigate the effects of HD on oxidative stress levels, the PI3K/AKT/mTOR signaling pathway, and mitophagy, exploring specific mechanisms related to PI3K/AKT/mTOR and Nrf2 signaling in mitigating skin photoaging. The findings aim to support the development and application of HD as a promising ingredient in anti-photoaging cosmetics.

## 2. Results

### 2.1. Chemical Contents of HD

Studies have shown that quercetin and chlorogenic acid possess mitochondrial protective and autophagy-promoting properties [29,30]. To determine their content in HD, we used high-performance liquid chromatography (HPLC) under optimized separation conditions tailored to each compound.

**Figure 1:**
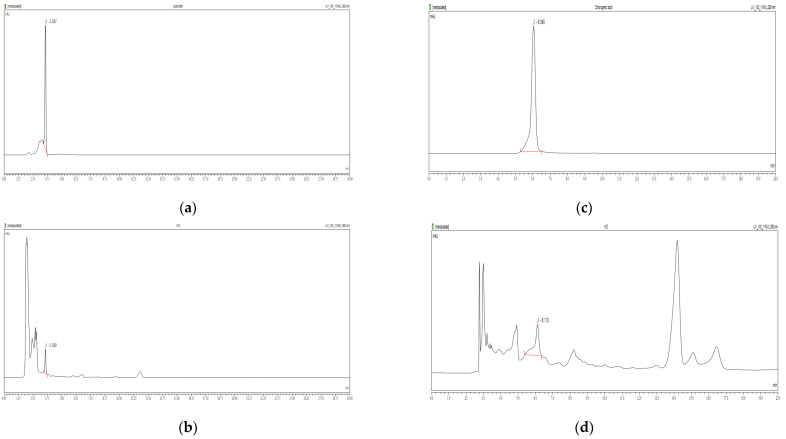
HPLC chromatograms of quercetin standards (**a**) and HD (**b**) at 360 nm, and chlorogenic acid standards (**c**) and HD (**d**) at 300 nm were obtained using a Discovery C18 column.

Figure 1a shows the retention time for quercetin standard as approximately 6.040 min. In Figure 1b, quercetin was detected in the HD sample with a calculated content of 0.41%. Similarly, Figure 1c shows the retention time for chlorogenic acid standard as 3.587 min, while Figure 1d identifies chlorogenic acid in the HD sample at 3.590 min, with a calculated content of 0.25%.

### 2.2. Antioxidant Effect of HD

Antioxidants can enhance autophagy, remove damaged cellular components, and reduce oxidative stress [31,32]. Therefore, we evaluated the antioxidant activity of HD by measuring its total flavonoid and total phenolic content, as well as its 2,2-diphenyl-1-picrylhydrazyl (DPPH) and 2,2′-azino-bis(3-ethylbenzothiazoline-6-sulfonic acid) (ABTS) radical scavenging capacities [33]. Ascorbic acid (AA), renowned for its antioxidant properties, was used as the positive control group.

**Figure 2:**
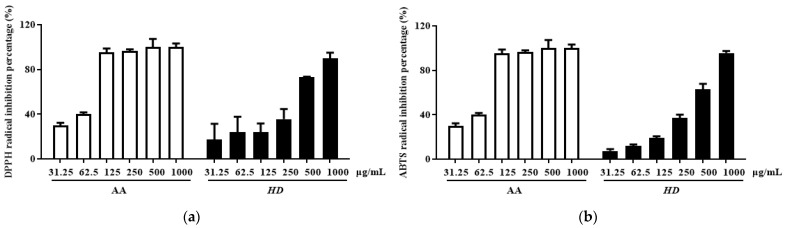
HD scavenging capacity for DPPH radicals (**a**) and ABTS radicals (**b**). Data are expressed as the mean ± SD from three independent replicates. AA represents ascorbic acid as a positive control group, and HD refers to *Hedyotis diffusa*.

Phenolic and flavonoid compounds are typical antioxidant components in natural products [34,35]. In HD, the concentrations of these compounds were determined to be 26.47 ± 1.16 mg gallic acid/g extract and 69.62 ± 0.91 mg quercetin/g extract, respectively. Furthermore, HD exhibited significant radical scavenging activity in a concentration-dependent manner. The IC_50_ value for DPPH scavenging was 268.1 μg/mL, with a confidence interval of 147.4–480.9 μg/mL (Figure 2a), while the IC_50_ value for ABTS scavenging was 319 μg/mL, with a confidence interval of 236.3–426.2 μg/mL (Figure 2b). These results indicate that HD possesses remarkable antioxidant activity.

### 2.3. Effects of HD on ROS Levels in HaCaT Keratinocytes Post-UVB Treatment

**Figure 3:**
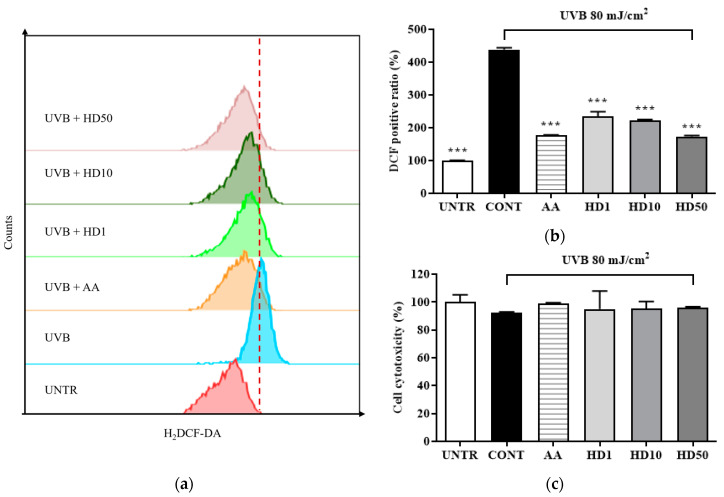
Flow cytometry (FACs) results for intracellular ROS levels detected using the 2′,7′-dichloro fluorescein diacetate (DCFH-DA) dye are shown in (**a**), with (**b**) presenting the quantified data as a histogram. Cell viability following HD treatment is shown in (**c**). Data are expressed as the mean ± SD from three independent replicates. *** *p*-value < 0.001 compared to the UVB-only treatment cells. AA represents 10 μM ascorbic acid as a positive control group, and HD1, 10, and 50 refer to *Hedyotis diffusa* at concentrations of 1, 10, and 50 μg/mL.

ROS are critical markers of mitochondrial dysfunction, and their levels typically increase significantly during mitochondrial damage [36,37]. Elevated ROS not only results from mitochondrial dysfunction but also exacerbates it further, triggering apoptotic pathways [38]. In Figure 3b, UVB treatment led to a substantial increase in intracellular ROS levels, reaching 338% of the control group, indicating severe mitochondrial damage.

In contrast, HD treatment effectively suppressed the UVB-induced abnormal increase in ROS levels, demonstrating remarkable antioxidant activity. At a concentration of 50 μg/mL, HD reduced ROS levels by 50%, an effect comparable to that of the positive control, ascorbic acid. These findings suggest that HD combats UVB-induced mitochondrial damage by effectively inhibiting intracellular ROS accumulation.

### 2.4. Effects of HD on Mitochondrial Membrane Function in HaCaT Keratinocytes Post-UVB Treatment

**Figure 4:**
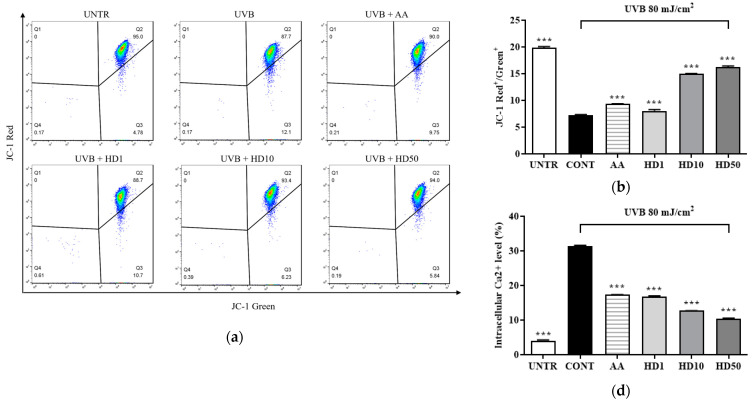

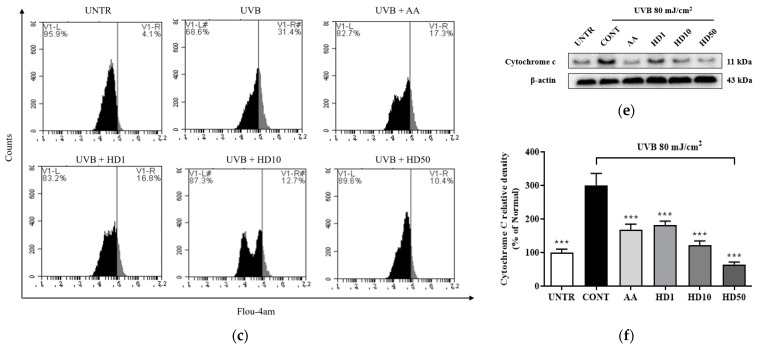
FACs results of intracellular MMP levels (**a**), with (**b**) showing the quantified data as a histogram. FACs results of intracellular Ca^2+^ levels (**c**), and (**d**) provide the corresponding quantified data in histogram form. Western blot result of the effect of HD on extramitochondrial cytochrome C (**e**), quantified by histogram (**f**). Data are expressed as the mean ± SD from three independent replicates. *** *p*-value < 0.001 compared to the UVB-only treatment group. AA represents 10 μM Ascorbic acid as a positive control group, and HD1, 10, and 50 refer to *Hedyotis diffusa* at concentrations of 1, 10, and 50 μg/mL.

UVB-induced ROS accumulation leads to a loss of mitochondrial membrane potential (MMP) and increased outer membrane permeability, facilitating the release of pro-apoptotic factors such as cytochrome C [36,39]. Excessive ROS also disrupts calcium homeostasis, causing abnormal elevations in cytoplasmic calcium concentrations and triggering calcium overload [40]. This overload further exacerbates mitochondrial dysfunction by activating the mitochondrial permeability transition pore [41]. As shown in Figure 4a,b, UVB treatment resulted in a significant 64% reduction in MMP, indicating severe mitochondrial damage. HD demonstrated a pronounced protective effect on MMP stability, with 50 μg/mL HD restoring MMP to 124% of the pre-UVB treatment levels, surpassing the protective effect of ascorbic acid.

Furthermore, UVB-induced mitochondrial membrane damage disrupted intracellular calcium homeostasis, leading to a 665% increase in cytoplasmic Ca^2+^ levels (Figure 4c,d). Although ascorbic acid partially reduced the UVB-induced elevation in cytoplasmic Ca^2+^ levels, 50 μg/mL HD achieved a remarkable 1.5-fold greater suppression, highlighting its superior regulatory capacity.

Additionally, as shown in Figure 4f, UVB exposure increased mitochondrial outer membrane permeability, resulting in a 200% elevation in cytosolic cytochrome C levels. HD significantly inhibited cytochrome c release, with 50 μg/mL HD reducing cytosolic cytochrome C levels by 78.5%. These findings suggest that HD effectively mitigates UVB-induced mitochondrial damage by stabilizing MMP, regulating calcium homeostasis, and inhibiting cytochrome C release.

### 2.5. Effects of HD on the Bax/Bcl-2 Ratio in HaCaT Keratinocytes Post-UVB Treatment

**Figure 5:**
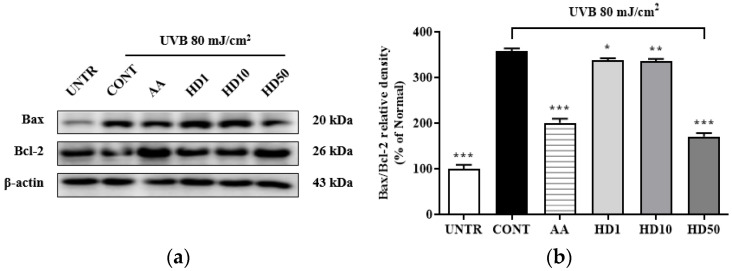
Western blot results of the effects of HD on the Bax/Bcl-2 ratio are shown in (**a**), with quantified displayed in histograms for Bax/Bcl-2 (**b**). Data are expressed as the mean ± SD from three independent replicates. * *p*-value < 0.05, ** *p*-value < 0.01, and *** *p*-value < 0.001 compared to the UVB-only treatment group. AA represents 10 μM ascorbic acid as a positive control group, and HD1, 10, and 50 refer to *Hedyotis diffusa* at concentrations of 1, 10, and 50 μg/mL.

Elevation of the Bax/Bcl-2 ratio is a critical marker of mitochondrial damage [42], which enhances mitochondrial outer membrane permeability and induces the opening of the permeability transition pore, thereby exacerbating membrane potential loss and the release of pro-apoptotic factors, ultimately activating the mitochondria-mediated apoptotic pathway [43]. As shown in Figure 5b, UVB treatment increased the Bax/Bcl-2 ratio by 258%, while 50 μg/mL HD treatment significantly reduced it by 52%. In comparison, ascorbic acid showed a reduction of 43.9%. However, low concentrations of HD at 1 μg/mL and 10 μg/mL exhibited negligible effects. These findings suggest that HD effectively suppressed mitochondrial damage and the further propagation of apoptotic signaling.

### 2.6. Effects of HD on the PINK/PARK2 and PI3K/AKT/mTOR Signaling Pathway in HaCaT Keratinocytes Post-UVB Treatment

**Figure 6:**
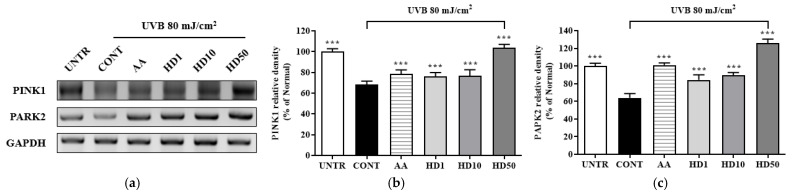

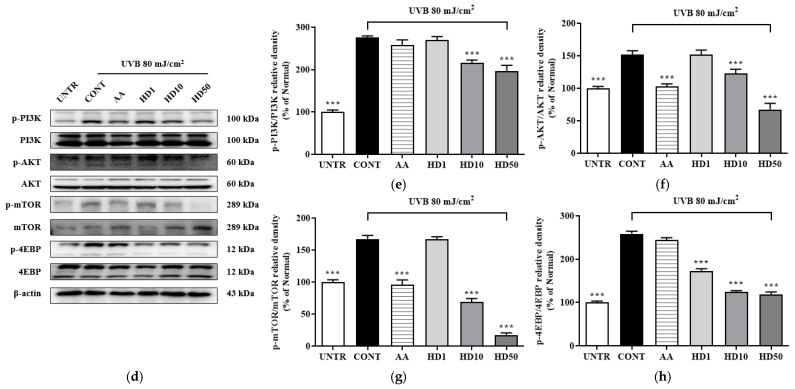
RT-PCR results of the effects of HD on the PINK1/PARK2 signaling pathway are shown in (**a**), with quantified displayed in histograms for PINK1 (**b**), and PARK2 (**c**). Western blot results of the effects of HD on the PI3K/AKT/mTOR signaling pathway are shown in (**d**), with quantified phosphorylation levels displayed in histograms for p-PI3K (**e**), p-AKT (**f**), p-mTOR (**g**), and p-4EBP (**h**). Data are expressed as the mean ± SD from three independent replicates. *** *p*-value < 0.001 compared to the UVB-only treatment group. AA represents 10 μM ascorbic acid as a positive control group, and HD1, 10, and 50 refer to *Hedyotis diffusa* at concentrations of 1, 10, and 50 μg/mL.

Studies have shown that impaired mitophagy caused by the inhibition of the PINK1/PARK2 pathway is a key mechanism underlying UVB-induced cellular damage [44,45]. PINK1 and PARK2, as critical regulators of mitophagy, maintain mitochondrial quality by selectively clearing damaged mitochondria [46]. Following UVB treatment of HaCaT keratinocytes, the expression levels of PINK1 and PARK2 were reduced by 31% and 36%, respectively (Figure 6a–c), disrupting mitophagy and leading to the accumulation of dysfunctional mitochondria. Treatment with ascorbic acid partially restored PINK1 and PARK2 expressions by 52% and 96%, respectively. In contrast, 50 μg/mL HD exhibited a more robust restorative effect, increasing PINK1 and PARK2 expression by 3.49-fold and 1.67-fold, respectively, compared to ascorbic acid.

The accumulation of dysfunctional mitochondria caused by UVB further elevated intracellular ROS levels [47]. The resulting oxidative stress abnormally activated the PI3K/AKT/mTOR signaling pathway, which inhibited the initiation of autophagy [48]. UVB exposure significantly increased the phosphorylation levels of PI3K, AKT, mTOR, and 4EBP1 (Figure 6d–h), indicating hyperactivation of this pathway. HD treatment effectively suppressed this activation, reducing the phosphorylation levels of PI3K, AKT, mTOR, and 4EBP1 by 28%, 55%, 53%, and 89%, respectively, outperforming ascorbic acid.

These findings suggest that UVB disrupts autophagy by impairing PINK1/PARK2 expression and indirectly promoting the activation of the PI3K/AKT/mTOR pathway. HD mitigates these effects, restoring autophagic balance and cellular homeostasis.

### 2.7. Effect of HD on Oxygen Consumption Rate (OCR) in HaCaT Keratinocytes Post-UVB Treatment

UVB-induced accumulation of damaged mitochondria reduces the efficiency of the electron transport chain (ETC), leading to significant declines in basal respiration and maximal respiratory capacity [49,50]. Furthermore, damaged mitochondria fail to synthesize ATP efficiently, resulting in a decrease in ATP-linked respiration [36]. These parameters in OCR analysis indirectly reflect mitophagy efficiency, as mitophagy maintains respiratory function and cellular homeostasis by removing damaged mitochondria [45].

In OCR measurements, oligomycin is used to inhibit ATP synthase, allowing for the evaluation of mitochondrial energy production efficiency and membrane integrity [51]. FCCP, an uncoupling agent, disrupts the proton gradient and forces ETC to operate at its maximum rate, enabling the assessment of maximal respiratory capacity and spare respiratory capacity. These metrics reflect the potential for mitochondrial functional recovery [52]. The combination of rotenone and antimycin A completely inhibits ETC activity, providing a baseline measurement of non-mitochondrial oxygen consumption [53].

**Figure 7:**
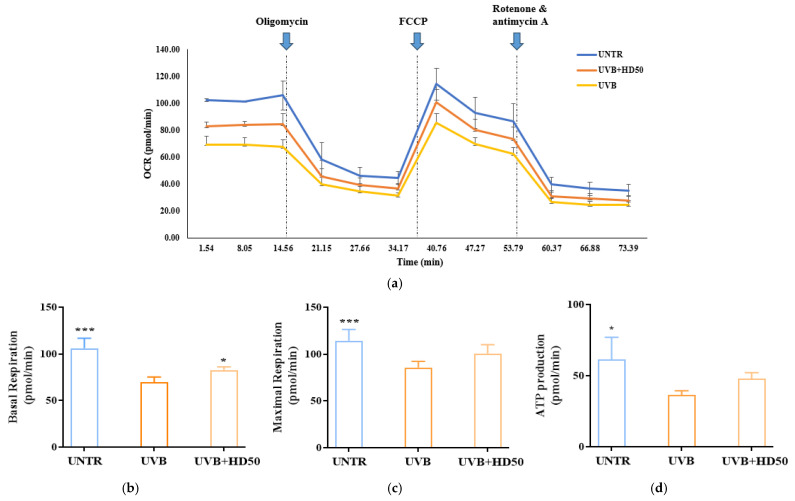
Seahorse XFp Analyzer measurements of mitochondrial respiration in cells treated with UVB and HD50 (**a**). Basal respiration (**b**), maximal respiration (**c**), and ATP production (**d**). Data are expressed as the mean ± SD from three independent replicates. * *p*-value < 0.05, and *** *p*-value < 0.001 compared to the UVB-only treatment group. HD50 refers to *Hedyotis diffusa* at concentrations of 50 μg/mL.

As shown in Figure 7a,b, UVB treatment reduced basal respiration and maximal respiratory capacity by 34% and 24%, respectively. However, treatment with 50 μg/mL HD significantly reversed these decreases, restoring basal respiration and maximal respiratory capacity by 18% and 17%, respectively. Additionally, ATP production, which was reduced by UVB treatment (Figure 7d), recovered by 31% following HD treatment. These data indicate that UVB-induced damage significantly impairs basal respiration, maximal respiratory capacity, and ATP production, while HD treatment effectively restores these parameters, partially reversing UVB-induced mitochondrial dysfunction. This restoration is likely closely associated with HD’s ability to promote mitophagy and enhance mitochondrial quality control.

### 2.8. Effects of HD on HaCaT Keratinocytes Cycle Post-UVB Treatment

**Figure 8:**
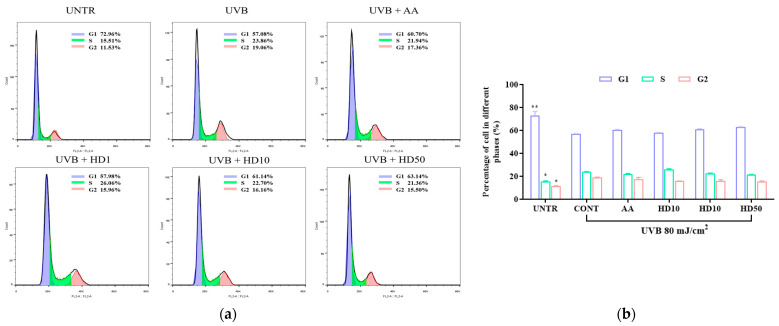
FACs analysis results of cell cycle (**a**), showing the quantified data as a histogram (**b**). Data are expressed as the mean ± SD from three independent replicates. * *p*-value < 0.05, and ** *p*-value < 0.01 compared to the UVB-only treatment group. AA represents 10 μM ascorbic acid as a positive control group, HD1, 10, and 50 refer to *Hedyotis diffusa* at concentrations of 1, 10, and 50 μg/mL.

UVB exacerbates HaCaT keratinocyte cycle arrest through impaired autophagy, driving keratinocytes into a senescent state [54]. Senescent keratinocytes contribute to photoaging by secreting the senescence-associated secretory phenotype [55]. We analyzed keratinocyte cycle progression to assess whether HD can counteract UVB-induced photoaging by modulating the keratinocyte cycle via autophagy activation. As shown in Figure 8a,b, UVB treatment increased the proportion of keratinocytes in the S and G2 phases by 8.5% and 7.53%, respectively, indicating significant G2/M phase arrest. While low concentrations of HD showed limited efficacy in reversing this arrest, 50 μg/mL of HD effectively promoted keratinocyte cycle progression, with a slightly greater effect than ascorbic acid.

### 2.9. Effects of HD on Apoptosis-Related in HaCaT Keratinocytes Post-UVB Treatment

**Figure 9:**
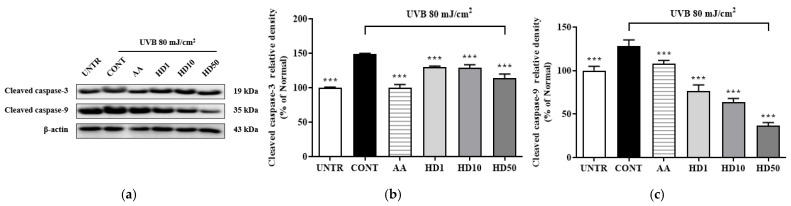
Western blot results of the effects of HD on the apoptosis-related proteins are shown in (**a**), with quantified displayed in histograms for cleaved caspase-3 (**b**), and cleaved caspase-9 (**c**). Data are expressed as the mean ± SD from three independent replicates. *** *p*-value < 0.001 compared to the UVB-only treatment group. AA represents 10 μM ascorbic acid as a positive control group, and HD1, 10, and 50 refer to *Hedyotis diffusa* at concentrations of 1, 10, and 50 μg/mL.

To determine whether the activation of autophagy by HD contributes to the restoration of cellular function by suppressing UVB-induced apoptosis, we examined the expression of apoptosis-related proteins, cleaved caspase-3 and cleaved caspase-9. As shown in Figure 9, UVB treatment significantly increased cleaved caspase-3 and cleaved caspase-9 levels by 258% and 48%, respectively. However, treatment with 50 μg/mL HD effectively reduced these levels by 52% and 71%, respectively. These results suggest that HD mitigates UVB-induced apoptosis, potentially through the activation of autophagy.

### 2.10. Effects of HD on the Nrf2/ARE Signaling Pathway in HaCaT Keratinocytes Post-UVB Treatment

**Figure 10:**
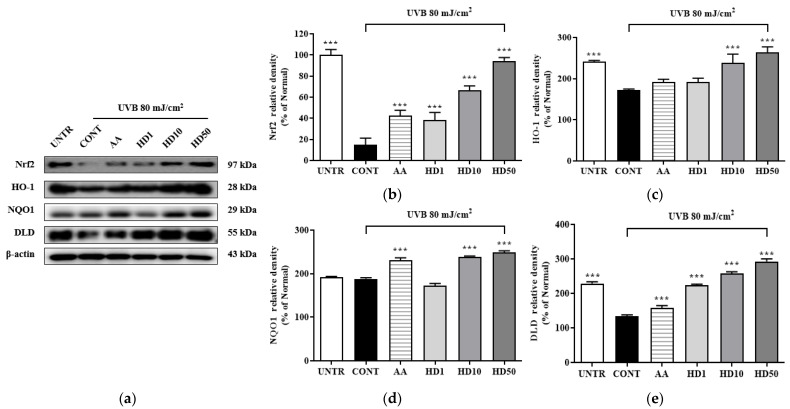
Western blot results of the effects of HD on the Nrf2 signaling pathway are shown in (**a**), with quantified displayed in histograms for Nrf2 (**b**), HO-1 (**c**), NQO1 (**d**), and DLD (**e**). Data are expressed as the mean ± SD from three independent replicates. *** *p*-value < 0.001 compared to the UVB-only treatment group. AA represents 10 μM ascorbic acid as a positive control group, and HD1, 10, and 50 refer to *Hedyotis diffusa* at concentrations of 1, 10, and 50 μg/mL.

The activation of Nrf2 plays a critical role in upregulating autophagy-related genes. To determine whether HD-induced autophagy involves the Nrf2 signaling pathway, we analyzed the expression of Nrf2/ARE-associated proteins. As shown in Figure 10, UVB exposure significantly suppressed Nrf2 pathway activity, reducing Nrf2 expression by 84% and the downstream antioxidant enzymes HO-1, NQO1, and DLD by 68%, 4%, and 95%, respectively. Notably, HD treatment not only reversed these reductions but also enhanced the expression of Nrf2, HO-1, NQO1, and DLD by 525%, 52%, 26%, and 119%, respectively, at a concentration of 50 μg/mL. These findings suggest that Nrf2/ARE plays a supportive role in the HD-mediated promotion of autophagy.

## 3. Discussion

Medicinal plants exhibit significant protective effects against UVB-induced skin photoaging by leveraging multi-target advantages through a multi-pathway regulation [56]. For instance, *Glycyrrhiza glabra*, *Lithospermum erythrorhizon*, and *Angelica gigas* are widely utilized as cosmetic ingredients [57,58,59]. However, current developments in anti-photoaging agents focus primarily on anti-inflammatory, antioxidant, and collagen regulation effects, with limited attention to mitochondrial regulation, which is closely related to aging [19]. This study aimed to determine whether HD can counteract UVB-induced photoaging by modulating the mitophagy of dysfunctional mitochondria in UVB-treated HaCaT keratinocytes to explore the potential of HD as a candidate for anti-aging cosmetic development.

Mitochondria are the primary energy source for cells, so any dysfunction directly impacts cellular vitality and tissue function, making mitochondrial health crucial in age-related diseases [10,60]. Research by Li et al. demonstrated that UVB exposure increases mitochondrial quantity but significantly reduces efficiency, suggesting that UVB inhibits mitophagy [44]. The mitochondrial ETC is composed of four main complexes (I–IV) located on the inner mitochondrial membrane, generating cellular energy through electron transfer [10,61]. However, UVB-induced ROS damages complexes I and III, reduces membrane potential, progressively exhausts mitochondrial function, and causes abnormal calcium accumulation and release [36,37]. Meanwhile, cytochrome c translocates into the cytoplasm, activating caspase-9 and initiating an apoptotic cascade, ultimately promoting cell death [62]. Excessive apoptosis can heighten the risk of skin cancer [44]. Intervening with mitophagy at mid-stage and accelerating the clearance of damaged mitochondria can help prevent programmed cell death [46,63]. Figure 4 shows that HD effectively maintains mitochondrial membrane potential, reduces proton leakage, and restores normal energy metabolism (Figure 4a–d) while lowering cytochrome c release (Figure 4f) and inhibiting apoptosis (Figure 9). This suggests HD is an effective mitochondrial health stabilizer.

Based on the evidence supporting HD’s role in maintaining mitochondrial health, we further explored its underlying mechanism. Studies indicate that *Artemisia* extract restores mitophagy by activating the PINK1/Parkin expression [64,65]. When mitochondria are damaged, and membrane potential decreases, PINK1 accumulates on the outer membrane and recruits Parkin (an E3 ubiquitin ligase) [10], marking damaged mitochondria for autophagic clearance. UVB exposure, however, suppresses PINK1/Parkin expression in HaCaT keratinocytes, blocking mitophagy. Notably, Figure 6a–c shows that HD effectively activates PINK1/Parkin. Meanwhile, the PI3K/AKT/mTOR pathway functions as a switch in regulating mitophagy; when phosphorylated, it inhibits mitophagy. Upon mTOR inhibition, 4EBP binds to eIF4E, suppressing protein synthesis and redirecting cellular resources toward repairing and clearing damaged mitochondria, thereby promoting mitophagy [66,67]. In neurodegenerative diseases, this mechanism helps clear protein aggregates and slows disease progression [68]. Moreover, Figure 6d–h demonstrate that HD effectively suppresses UVB-induced activation of the PI3K/AKT/mTOR pathway. Compared to other plant extracts, HD exhibits a unique regulatory effect on these pathways. For instance, extracts like resveratrol are known to inhibit the PI3K/AKT/mTOR pathway [12], but they lack the robust dual activation of PINK1/Parkin observed with HD. This indicates that HD plays a dual regulatory role in both the PINK1/Parkin and PI3K/AKT/mTOR pathways, contributing to the maintenance of mitochondrial health.

Additionally, Nrf2 acts as an auxiliary regulatory pathway in autophagy. Research has shown that maltol promotes mitophagy and prevents neuronal apoptosis by activating Nrf2 to stimulate the PINK1/Parkin pathway [69]. Similarly, in hyperglycemia-induced tubular cell damage, Nrf2 enhances mitochondrial quality by facilitating mitophagy through PINK1/Parkin, reducing ROS accumulation and protecting cells from injury [70]. While no studies have explored Nrf2/PINK1/Parkin expression in UVB-treated HaCaT keratinocytes, our research aligns with this perspective. Figure 10 demonstrates that HD effectively activates the Nrf2 pathway in UVB-treated HaCaT keratinocytes, aiding in the clearance of damaged mitochondria, further indicating that Nrf2 plays a supportive role in HD-mediated autophagy activation.

**Figure 11:**
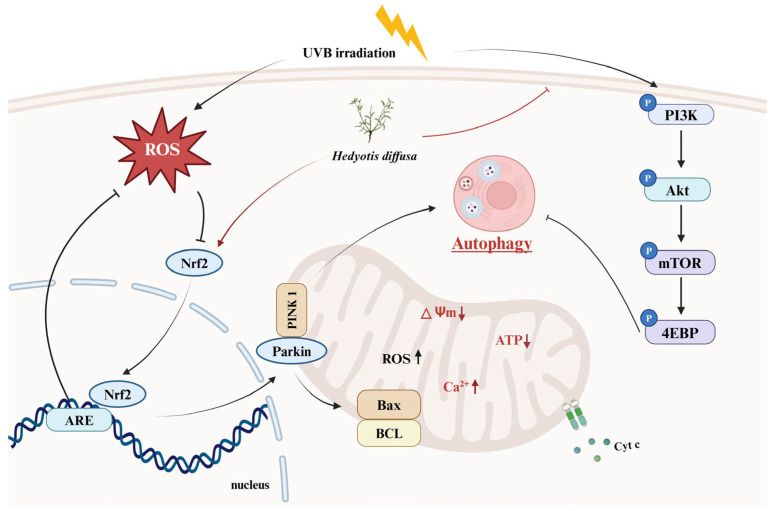
HD extract activates autophagy in HaCaT keratinocytes post-UVB treatment by regulating PI3K/AKT/mTOR and Nrf2.

Although we demonstrated the anti-photoaging effects of HD using in vitro cell models (Figure 11), these models cannot fully replicate the complexity of human skin. Notably, HaCaT cells, as an immortalized line with a P53 mutation, may not fully represent the behavior of normal keratinocytes. Therefore, further validation is needed using normal primary keratinocytes and human skin organ cultures to simulate physiological conditions better. Additionally, the bioavailability of HD’s active ingredients poses a significant challenge in cosmetic development [71]. Advanced delivery systems, such as nanoparticles, liposomes, or microemulsions, may be required to enhance its absorption and effectiveness [72].

## 4. Materials and Methods

### 4.1. HD Extractions

Dried HD was supplied by Bozhou Mingjie Biotechnology Co., Ltd. (Bozhou, China) During the preparation process, the crushed 100 g HD was immersed in a 1 L 30% (*v*/*v*) ethanol solution and stirred overnight. Subsequently, the mixture was filtered using a 5 µm filter paper (HYUNDAI MICRO, Anseong-si, Republic of Korea). The filtered product was then concentrated under pressure using a Low-Temperature Circulator CoolAce CCA-1112A (EYELA, Tokyo, Japan) and reduced to a powder through vacuum evaporation. The yield of the final sample was 27.6%. Currently, this HD extract is properly stored in the laboratory at Kyung Hee University Global Campus in Yongin, Republic of Korea.

### 4.2. HPLC

Both 1 mg/mL HD and the standard compounds (quercetin and chlorogenic acid) were dissolved in 100% methanol. The Dionex Chromeleon™ system (Thermo Fisher Scientific, Waltham, MA, USA), featuring a UVD100 detector and a P580 pump, was used for HPLC analysis. Chromatographic separation was performed on a Discovery C18 column (25 cm × 4.5 mm, 5 μm; Supelco, Inc., Bellefonte, PA, USA). The flow rate was set to 1.0 mL/min, with an injection volume of 30 μL, while the column was kept at 23 °C. A detailed description of the analytical methods is provided in Supplementary Table S1.

### 4.3. Measurement of Antioxidant Capacity

The contents of phenol and flavonoid compounds in HD were determined using the Folin–Ciocalteu method and nitrite colorimetric method, following previously described protocols [3]. Briefly, total phenol content was assessed by reacting HD with Folin–Ciocalteu reagent and Na_2_CO_3_ solution and measured at 760 nm using a FilterMax F5 microplate reader (Molecular Devices, San Francisco, CA, USA), with gallic acid as the standard. Total flavonoid content was measured via the nitrite colorimetric method [3], where HD was reacted with 10% AlCl_3_ solution and recorded at 510 nm, using quercetin as the standard for quantification.

HD’s antioxidant capacity was confirmed by its DPPH and ABTS radical scavenging activities. For the DPPH assay, 40 µL of HD at concentrations ranging from 31.25 to 1000 µg/mL was combined with 160 µL of 0.2 mM DPPH solution and incubated in the dark for 30 min, after which absorbance was read at 595 nm. For the ABTS assay, a solution of 2.5 mM ABTS and 1 mM 2,2′-azobis (2-amidinopropane) dihydrochloride (AAPH) in 150 mM sodium chloride was prepared [73]. HD and ABTS solutions were mixed in equal volumes and incubated in the dark for 10 min before being measured at 405 nm. Ascorbic acid served as positive control. The inhibition of DPPH and ABTS radicals by the samples was determined using the following formula:

(1) $$DPPH\;and\;ABTS\;radical\;scavenging\left(\%\right)=\frac{{OD}_{0}-{OD}_{X}}{{OD}_{0}}\times 100$$

where *OD*_0_ represents the optical density measured for the negative control, and *OD_X_* represents the optical density measured at different concentrations of HD or AA.

### 4.4. Cell Culture and Photoaging Model Establishment

HaCaT keratinocytes (ATCC, Manassas, VA, USA) were cultured at 37 °C in a 5% CO_2_ incubator using Dulbecco’s Modified Eagle Medium (DMEM) (Sigma-Aldrich, St. Louis, MO, USA) supplemented with 10% fetal bovine serum (Hyclone, Logan, UT, USA).

When the cells reached 80% confluence, HaCaT keratinocytes were treated with UVB irradiation at 80 mJ/cm^2^ using the Bio-Link BLX-312 system (Vilber Lourmat GmbH, Marne-la-Vallée, France) [73]. Following UVB exposure, the cells were incubated with serum-free DMEM diluted with HD samples or ascorbic acid. The treatment time depended on the specific experiment. The selection of HD concentrations was based on previous studies [74].

### 4.5. Cytotoxicity

HaCaT keratinocytes viability after HD treatment was assessed by incubating cells overnight, adding 0.1 mg/mL Thiazolyl Blue Tetrazolium Bromide (Sigma, St. Louis, MO, USA), and keeping them in the dark for 4 h to form formazan crystals. The crystals were dissolved in DMSO and measured at 570 nm [3].

### 4.6. FACs

Fluo-4 AM fluorescent probe was used to assess intracellular Ca^2+^ levels. After being treated for 24 h, cells were digested and collected using 0.25% trypsin-EDTA (TE) (Gibco, Carlsbad, CA, USA), washed twice, and resuspended in 1X PBS. These cells were incubated with 1 µM Fluo-4 AM (Invitrogen, Waltham, MA, USA) at 37 °C for 1 h. After incubation, the cells were washed and resuspended in 1X PBS and analyzed by BD Accuri C6 flow cytometer (BD Biosciences, Ann Arbor, MI, USA) using the FITC channel.

For ROS detection, after being treated for 24 h, cells were incubated with 30 µM DCFH-DA (Sigma) in the dark for 30 min, and the cells were harvested using 0.25% TE. Intracellular ROS levels were quantified using a FITC channel.

To evaluate the effect of HD on the cell cycle, cells were treated for 48 h and then fixed in 70% ethanol at 4 °C overnight after RNase A treatment (1.5 mg/L) and PI (BD Biosciences, Franklin Lakes, NJ, USA) staining. Cell cycle distribution was determined using the PE channel.

For MMP analysis, cells were treated for 48 h, then collected and washed with 0.25% TE. The cells were subsequently resuspended in 1X PBS and stained with 2.5 μg/mL JC-1 dye (Sigma) at 37 °C for 30 min. After staining, the cells were washed and resuspended in 1X PBS, followed by detection using both PE and FITC channels.

### 4.7. Measurement of the Cellular OCR

The cellular OCR assay was conducted with reference to previous studies [52]. Mitochondrial respiration was assessed using the Agilent Seahorse XF Cell Mito Stress Test Kit and the Seahorse XF HS Mini Analyzer (Agilent Technologies, Santa Clara, CA, USA). Cells were seeded into specialized culture plates, and after overnight incubation, the medium was replaced with the assay-specific medium provided in the kit. The plates were then incubated in a non-CO_2_ incubator at 37 °C for up to 30 min. Following incubation, oligomycin (1.5 µM), FCCP (0.5 µM), and a combination of rotenone and antimycin A (0.5 µM) were sequentially injected into different ports of the flux cartridge according to the manufacturer’s instructions.

(2) Basal Respiration=Initial OCR−Non−mitochondrial OCR

*Maximal Respiratory Capacity* = *OCR after FCCP treatment* − *Non-mitochondrial OCR* (3)

(4) ATP production=Basal OCR−OCR after oligomycin treatment

### 4.8. RT-PCR

After being treated for 24 h, RNA was extracted using TRIZOL reagent (Invitrogen Life Technologies, Carlsbad, CA, USA). For cDNA synthesis, 2 µg of total RNA was reverse transcribed with oligo-dT18 primers. PCR amplification was conducted using PCR premix (Bioneer Co., Daejeon, Republic of Korea). RT-PCR was conducted on a Veriti thermal cycler (Applied Biosystems, Foster City, CA, USA) with the following primer pairs: human PINK, forward 5′-CCC AAG CAA CTA GCC CCT C-3′, reverse 5′-GGC AGC ACA TCA GGG TAG TC-3′; human PARK2, forward 5′-CCC ACC TCT GAC AAG GAA ACA-3′, reverse 5′-TCG TGA ACA AAC TGC CGA TCA-3′; and human GAPDH, forward 5′-GAA GGT GAA GGT CGG AGT C-3′, reverse 5′-GAA GAT GGT GAT GGG ATT TC-3′. Each experiment was performed in triplicate with specificity optimized prior to experimentation.

### 4.9. Western Blotting

Cells were harvested using a cell scraper to collect cells after treatment for 3, 4, 6, and 9 h, then centrifuged at 12,000 rpm for 20 min. After removing the supernatant, we added 50 µL of RIPA buffer and lysed the cells at 4 °C for 10 min. Following lysis, they were centrifuged again to collect the supernatant, then protein concentration was quantified using the Pierce™ BCA Protein Assay Kit (Thermo Scientific, Rockford, IL, USA). Mitochondrial and cytoplasmic proteins were separated using the Mitochondrial Isolation Kit (Beyotime Institute of Biotechnology, Nantong, China) and the quantified samples were mixed with sample buffer and denatured at 95 °C. After SDS-PAGE, the proteins were transferred to an Immobilon-P PVDF membrane (Millipore, Burlington, MA, USA) using the wet transfer method. The membrane was blocked and incubated overnight with specific primary antibodies from Cell Signaling Technology (Beverly, MA, USA) and Santa Cruz Biotechnology (Santa Cruz, CA, USA). After washing with TBST buffer, samples were incubated with appropriate secondary antibodies from Bio-Rad (Hercules, CA, USA). Signals were detected using Dyne ECL Pico Plus (DYNEBIO, Seongnam, Republic of Korea) and results analyzed with UVI-1D v. 16.11a program (UVITEC, Warwickshire, UK).

### 4.10. Statistical Methods and Analysis

Data were assessed using FlowJo_V10.8.1 (Treestar, Ashland, OR, USA), GraphPad Prism 8 (GraphPad Software Inc., La Jolla, CA, USA), and ImageJ (https://imagej.net/ij/, National Institutes of Health, Bethesda, MD, USA). The presentation of the results involved the average ± SD from three independent experiments, subsequently analyzed using analysis of variance (ANOVA). * *p*-value < 0.05, ** *p*-value < 0.01, and *** *p*-value < 0.001.

## 5. Conclusions

This study indicates that HD is a promising cosmetic additive capable of effectively stabilizing mitochondrial health, thereby combating UVB-induced skin photoaging. HD modulates mitophagy through the PINK1/Parkin and PI3K/AKT/mTOR pathways, mitigating UVB-induced mitochondrial dysfunction and apoptosis in HaCaT keratinocytes.

## Data Availability

Data are contained within the article.

## Supplementary Materials

The following supporting information can be downloaded at: https://www.mdpi.com/article/10.3390/molecules30020261/s1, Table S1: The detailed elution conditions utilized for HPLC.

## References

[1] Chung J.H., Hanft V.N., Kang S. (2003). Aging and Photoaging. J. Am. Acad. Dermatol..

[2] Slominski A.T., Zmijewski M.A., Plonka P.M., Szaflarski J.P., Paus R. (2018). How UV Light Touches the Brain and Endocrine System Through Skin, and Why. Endocrinology.

[3] Kim H., Zheng Q., Oh S., Zheng S., Kim M., Yi T.-H. (2023). Anti-Photoaging Effect of Jawoongo via Regulating Nrf2/ARE and TGF-β/Smad Signaling in In Vitro Photoaging Model. Appl. Sci..

[4] Umar S.A., Tasduq S.A. (2020). Integrating DNA Damage Response and Autophagy Signalling Axis in Ultraviolet-B Induced Skin Photo-Damage: A Positive Association in Protecting Cells against Genotoxic Stress. RSC Adv..

[5] Song X., Narzt M.S., Nagelreiter I.M., Hohensinner P., Terlecki-Zaniewicz L., Tschachler E., Grillari J., Gruber F. (2017). Autophagy Deficient Keratinocytes Display Increased DNA Damage, Senescence and Aberrant Lipid Composition after Oxidative Stress in Vitro and in Vivo. Redox Biol..

[6] Wang M., Charareh P., Lei X., Zhong J.L. (2019). Autophagy: Multiple Mechanisms to Protect Skin from Ultraviolet Radiation-Driven Photoaging. Oxidative Med. Cell. Longev..

[7] Tang Z., Tong X., Huang J., Liu L., Wang D., Yang S. (2021). Research Progress of Keratinocyte-programmed Cell Death in UV-induced Skin Photodamage. Photoderm. Photoimm. Photomed..

[8] Ma J., Teng Y., Huang Y., Tao X., Fan Y. (2022). Autophagy Plays an Essential Role in Ultraviolet Radiation-Driven Skin Photoaging. Front. Pharmacol..

[9] Slominski R.M., Chen J.Y., Raman C., Slominski A.T. (2024). Photo-Neuro-Immuno-Endocrinology: How the Ultraviolet Radiation Regulates the Body, Brain, and Immune System. Proc. Natl. Acad. Sci. USA.

[10] He H., Xiong L., Jian L., Li L., Wu Y., Qiao S. (2022). Role of Mitochondria on UV-Induced Skin Damage and Molecular Mechanisms of Active Chemical Compounds Targeting Mitochondria. J. Photochem. Photobiol. B Biol..

[11] Wang Y., Wen X., Hao D., Zhou M., Li X., He G., Jiang X. (2019). Insights into Autophagy Machinery in Cells Related to Skin Diseases and Strategies for Therapeutic Modulation. Biomed. Pharmacother..

[12] Vikram A., Patel S.K., Singh A., Pathania D., Ray R.S., Upadhyay A.K., Dwivedi A. (2024). Natural Autophagy Activators: A Promising Strategy for Combating Photoaging. Phytomedicine.

[13] Cavinato M., Martic I., Wedel S., Pittl A., Koziel R., Weinmmüllner R., Schosserer M., Jenewein B., Bobbili M.R., Arcalis E. (2024). Elimination of Damaged Mitochondria during UVB-induced Senescence Is Orchestrated by NIX-dependent Mitophagy. Aging Cell.

[14] Oblong J.E., Bowman A., Rovito H.A., Jarrold B.B., Sherrill J.D., Black M.R., Nelson G., Kimball A.B., Birch-Machin M.A. (2020). Metabolic Dysfunction in Human Skin: Restoration of Mitochondrial Integrity and Metabolic Output by Nicotinamide (Niacinamide) in Primary Dermal Fibroblasts from Older Aged Donors. Aging Cell.

[15] Yang Z., Zeng B., Pan Y., Huang P., Wang C. (2018). Autophagy Participates in Isoliquiritigenin–Induced Melanin Degradation in Human Epidermal Keratinocytes through PI3K/AKT/mTOR Signaling. Biomed. Pharmacother..

[16] Yang J., Pi C., Wang G. (2018). Inhibition of PI3K/Akt/mTOR Pathway by Apigenin Induces Apoptosis and Autophagy in Hepatocellular Carcinoma Cells. Biomed. Pharmacother..

[17] Heras-Sandoval D., Pérez-Rojas J.M., Hernández-Damián J., Pedraza-Chaverri J. (2014). The Role of PI3K/AKT/mTOR Pathway in the Modulation of Autophagy and the Clearance of Protein Aggregates in Neurodegeneration. Cell. Signal..

[18] Zhai C., Cheng J., Mujahid H., Wang H., Kong J., Yin Y., Li J., Zhang Y., Ji X., Chen W. (2014). Selective Inhibition of PI3K/Akt/mTOR Signaling Pathway Regulates Autophagy of Macrophage and Vulnerability of Atherosclerotic Plaque. PLoS ONE.

[19] Yang H.-L., Chen S.-J., Yeh J.-T., Vadivalagan C., Chiu J.-H., Hseu J.-H., Hseu Y.-C. (2024). The Anti-Melanogenesis, Anti-Photoaging, and Anti-Inflammation of Coenzyme Q0, a Major Quinone Derivative from Antrodia Camphorata, through Antioxidant Nrf2 Signaling Pathways in UVA/B-Irradiated Keratinocytes. J. Funct. Foods.

[20] Chaiprasongsuk A., Panich U. (2022). Role of Phytochemicals in Skin Photoprotection via Regulation of Nrf2. Front. Pharmacol..

[21] Zhang J., Zheng Y., Hong B., Ma L., Zhao Y., Zhang S., Sun S., Ding Q., Wang Y., Liu W. (2022). Dihydroquercetin Composite Nanofibrous Membrane Prevents UVA Radiation-Mediated Inflammation, Apoptosis and Oxidative Stress by Modulating MAPKs/Nrf2 Signaling in Human Epidermal Keratinocytes. Biomed. Pharmacother..

[22] Liu C., Vojnovic D., Kochevar I.E., Jurkunas U.V. (2016). UV-A Irradiation Activates Nrf2-Regulated Antioxidant Defense and Induces P53/Caspase3-Dependent Apoptosis in Corneal Endothelial Cells. Investig. Ophthalmol. Vis. Sci..

[23] Liu W., Yan F., Xu Z., Chen Q., Ren J., Wang Q., Chen L., Ying J., Liu Z., Zhao J. (2022). Urolithin A Protects Human Dermal Fibroblasts from UVA-Induced Photoaging through NRF2 Activation and Mitophagy. J. Photochem. Photobiol. B Biol..

[24] Lu F., Zhou Q., Liang M., Liang H., Yu Y., Li Y., Zhang Y., Lu L., Zheng Y., Hao J. (2024). α-Arbutin Ameliorates UVA-Induced Photoaging through Regulation of the SIRT3/PGC-1α Pathway. Front. Pharmacol..

[25] Wang K.-H., Lin R.-D., Hsu F.-L., Huang Y.-H., Chang H.-C., Huang C.-Y., Lee M.-H. (2006). Cosmetic Applications of Selected Traditional Chinese Herbal Medicines. J. Ethnopharmacol..

[26] Zhang R., Ma C., Wei Y., Wang X., Jia J., Li J., Li K., Cao G., Yang P. (2021). Isolation, Purification, Structural Characteristics, Pharmacological Activities, and Combined Action of Hedyotis Diffusa Polysaccharides: A Review. Int. J. Biol. Macromol..

[27] Chen R., He J., Tong X., Tang L., Liu M. (2016). The Hedyotis Diffusa Willd. (*Rubiaceae*): A Review on Phytochemistry, Pharmacology, Quality Control and Pharmacokinetics. Molecules.

[28] Park J.H., Whang W.K. (2020). Bioassay-Guided Isolation of Anti-Alzheimer Active Components from the Aerial Parts of Hedyotis Diffusa and Simultaneous Analysis for Marker Compounds. Molecules.

[29] De Oliveira M.R., Nabavi S.M., Braidy N., Setzer W.N., Ahmed T., Nabavi S.F. (2016). Quercetin and the Mitochondria: A Mechanistic View. Biotechnol. Adv..

[30] Gao L.-J., Dai Y., Li X.-Q., Meng S., Zhong Z.-Q., Xu S.-J. (2021). Chlorogenic Acid Enhances Autophagy by Upregulating Lysosomal Function to Protect against SH-SY5Y Cell Injury Induced by H_2_ O_2_. Exp. Ther. Med..

[31] Yun H.R., Jo Y.H., Kim J., Shin Y., Kim S.S., Choi T.G. (2020). Roles of Autophagy in Oxidative Stress. Int. J. Mol. Sci..

[32] Mizushima N., Levine B. (2010). Autophagy in Mammalian Development and Differentiation. Nat. Cell Biol..

[33] Brand-Williams W., Cuvelier M.E., Berset C. (1995). Use of a Free Radical Method to Evaluate Antioxidant Activity. LWT-Food Sci. Technol..

[34] Rice-Evans C., Miller N., Paganga G. (1997). Antioxidant Properties of Phenolic Compounds. Trends Plant Sci..

[35] Panche A.N., Diwan A.D., Chandra S.R. (2016). Flavonoids: An Overview. J. Nutr. Sci..

[36] Murphy M.P. (2009). How Mitochondria Produce Reactive Oxygen Species. Biochem. J..

[37] Zorov D.B., Juhaszova M., Sollott S.J. (2014). Mitochondrial Reactive Oxygen Species (ROS) and ROS-Induced ROS Release. Physiol. Rev..

[38] Chandra D., Liu J.-W., Tang D.G. (2002). Early Mitochondrial Activation and Cytochrome c Up-Regulation during Apoptosis. J. Biol. Chem..

[39] Ott M., Gogvadze V., Orrenius S., Zhivotovsky B. (2007). Mitochondria, Oxidative Stress and Cell Death. Apoptosis.

[40] Duchen M.R. (2000). Mitochondria and Calcium: From Cell Signalling to Cell Death. J. Physiol..

[41] Halestrap A.P., Richardson A.P. (2015). The Mitochondrial Permeability Transition: A Current Perspective on Its Identity and Role in Ischaemia/Reperfusion Injury. J. Mol. Cell. Cardiol..

[42] Danial N.N., Korsmeyer S.J. (2004). Cell Death. Cell.

[43] Youle R.J., Strasser A. (2008). The BCL-2 Protein Family: Opposing Activities That Mediate Cell Death. Nat. Rev. Mol. Cell Biol..

[44] Li C., Zhu Y., Liu W., Xiang W., He S., Hayashi T., Mizuno K., Hattori S., Fujisaki H., Ikejima T. (2023). Impaired Mitophagy Causes Mitochondrial DNA Leakage and STING Activation in Ultraviolet B-Irradiated Human Keratinocytes HaCaT. Arch. Biochem. Biophys..

[45] Pickles S., Vigié P., Youle R.J. (2018). Mitophagy and Quality Control Mechanisms in Mitochondrial Maintenance. Curr. Biol..

[46] Lazarou M., Sliter D.A., Kane L.A., Sarraf S.A., Wang C., Burman J.L., Sideris D.P., Fogel A.I., Youle R.J. (2015). The Ubiquitin Kinase PINK1 Recruits Autophagy Receptors to Induce Mitophagy. Nature.

[47] Li N., Ragheb K., Lawler G., Sturgis J., Rajwa B., Melendez J.A., Robinson J.P. (2003). Mitochondrial Complex I Inhibitor Rotenone Induces Apoptosis through Enhancing Mitochondrial Reactive Oxygen Species Production. J. Biol. Chem..

[48] Jung C.H., Ro S.-H., Cao J., Otto N.M., Kim D.-H. (2010). mTOR Regulation of Autophagy. FEBS Lett..

[49] Brand M.D., Nicholls D.G. (2011). Assessing Mitochondrial Dysfunction in Cells. Biochem. J..

[50] Nunnari J., Suomalainen A. (2012). Mitochondria: In Sickness and in Health. Cell.

[51] Dranka B.P., Benavides G.A., Diers A.R., Giordano S., Zelickson B.R., Reily C., Zou L., Chatham J.C., Hill B.G., Zhang J. (2011). Assessing Bioenergetic Function in Response to Oxidative Stress by Metabolic Profiling. Free Radic. Biol. Med..

[52] Kim G., Han D.-W., Lee J.H. (2023). The Cytoprotective Effects of Baicalein on H_2_O_2_-Induced ROS by Maintaining Mitochondrial Homeostasis and Cellular Tight Junction in HaCaT Keratinocytes. Antioxidants.

[53] Zhang J., Ney P.A. (2009). Role of BNIP3 and NIX in Cell Death, Autophagy, and Mitophagy. Cell Death Differ..

[54] Zhang J.-A., Luan C., Huang D., Ju M., Chen K., Gu H. (2020). Induction of Autophagy by Baicalin Through the AMPK-mTOR Pathway Protects Human Skin Fibroblasts from Ultraviolet B Radiation-Induced Apoptosis. Drug Des. Dev. Ther..

[55] Wang A.S., Dreesen O. (2018). Biomarkers of Cellular Senescence and Skin Aging. Front. Genet..

[56] Peng X., Ma Y., Yan C., Wei X., Zhang L., Jiang H., Ma Y., Zhang S., Xing M., Gao Y. (2024). Mechanism, Formulation, and Efficacy Evaluation of Natural Products for Skin Pigmentation Treatment. Pharmaceutics.

[57] Cerulli A., Masullo M., Montoro P., Piacente S. (2022). Licorice (*Glycyrrhiza glabra*, *G. uralensis*, and *G. inflata*) and Their Constituents as Active Cosmeceutical Ingredients. Cosmetics.

[58] Chang M.-J., Huang H.-C., Chang H.-C., Chang T.-M. (2008). Cosmetic Formulations Containing Lithospermum Erythrorhizon Root Extract Show Moisturizing Effects on Human Skin. Arch. Dermatol. Res..

[59] Kang J., Cho H., Choi H., Lee I. (2023). Anti-wrinkle Properties of *Angelica gigas* Nakai Root Extracts Using Mineral-rich Water. J. Cosmet. Dermatol..

[60] Sreedhar A., Aguilera-Aguirre L., Singh K.K. (2020). Mitochondria in Skin Health, Aging, and Disease. Cell Death Dis..

[61] Zhu Y., Xiang W., He S., San Z., Liu W., Wu J., Hayashi T., Mizuno K., Hattori S., Fujisaki H. (2024). Collagen I Protects Human Keratinocytes HaCaT against UVB Injury via Restoring PINK1/Parkin-Mediated Mitophagy. Arch. Biochem. Biophys..

[62] Elmore S. (2007). Apoptosis: A Review of Programmed Cell Death. Toxicol. Pathol..

[63] Youle R.J., Narendra D.P. (2011). Mechanisms of Mitophagy. Nat. Rev. Mol. Cell Biol..

[64] Zhou X., Wang J., Yu L., Qiao G., Qin D., Law B.Y.K., Ren F., Wu J., Wu A. (2024). Mitophagy and cGAS–STING Crosstalk in Neuroinflammation. Acta Pharm. Sin. B.

[65] Liang H., Ma Z., Zhong W., Liu J., Sugimoto K., Chen H. (2024). Regulation of Mitophagy and Mitochondrial Function: Natural Compounds as Potential Therapeutic Strategies for Parkinson’s Disease. Phytother. Res..

[66] Saxton R.A., Sabatini D.M. (2017). mTOR Signaling in Growth, Metabolism, and Disease. Cell.

[67] Kim J., Kundu M., Viollet B., Guan K.-L. (2011). AMPK and mTOR Regulate Autophagy through Direct Phosphorylation of Ulk1. Nat. Cell Biol..

[68] Yu Q., Zhang R., Li T., Yang L., Zhou Z., Hou L., Wu W., Zhao R., Chen X., Yao Y. (2023). Correction to: Mitochondrial Hydrogen Peroxide Activates PTEN and Inactivates Akt Leading to Autophagy Inhibition-Dependent Cell Death in Neuronal Models of Parkinson’s Disease. Mol. Neurobiol..

[69] Mao Y., Du J., Chen X., Al Mamun A., Cao L., Yang Y., Mubwandarikwa J., Zaeem M., Zhang W., Chen Y. (2022). Maltol Promotes Mitophagy and Inhibits Oxidative Stress via the Nrf2/PINK1/Parkin Pathway after Spinal Cord Injury. Oxidative Med. Cell. Longev..

[70] Xiao L., Xu X., Zhang F., Wang M., Xu Y., Tang D., Wang J., Qin Y., Liu Y., Tang C. (2017). The Mitochondria-Targeted Antioxidant MitoQ Ameliorated Tubular Injury Mediated by Mitophagy in Diabetic Kidney Disease via Nrf2/PINK1. Redox Biol..

[71] Santos A.C., Morais F., Simões A., Pereira I., Sequeira J.A.D., Pereira-Silva M., Veiga F., Ribeiro A. (2019). Nanotechnology for the Development of New Cosmetic Formulations. Expert. Opin. Drug Deliv..

[72] Akhtar N., Singh V., Yusuf M., Khan R.A. (2020). Non-Invasive Drug Delivery Technology: Development and Current Status of Transdermal Drug Delivery Devices, Techniques and Biomedical Applications. Biomed. Eng. Biomed. Tech..

[73] Lee S., Oh S., Zheng Q., Zheng S., Kim M., Park S., Choi W., Yin C.S., Yi T. (2024). Photoprotective Effects of *Lithospermum erythrorhizon* and *Pueraria lobata* Extracts on UVB-induced Photoaging: A Study on Skin Barrier Protection. Photoderm. Photoimm. Photomed..

[74] Kim M., Ha L.-K., Oh S., Fang M., Zheng S., Bellere A.D., Jeong J., Yi T.-H. (2022). Antiphotoaging Effects of Damiana (*Turnera diffusa*) Leaves Extract via Regulation AP-1 and Nrf2/ARE Signaling Pathways. Plants.

