# Supplementary material for: Autophagy-Enhancing Properties of Hedyotis diffusa Extracts in HaCaT Keratinocytes: Potential as an Anti-Photoaging Cosmetic Ingredient
Source: Molecules. 2025 Jan 10;30(2):261. doi: 10.3390/molecules30020261 (PMC11767327; doi:10.3390/molecules30020261)
Supplement: Supplementary file 1 [file molecules-30-00261-s001.zip › molecules-3367006-supplementary.pdf]

Table S1. HPLC method for the identification of HD extract

	Quercetin	Chlorogenic acid
**Mobile phase A**	0.1% Formic acid in DW (v/v)	
**Mobile phase B**	0.1% Formic acid in acetonitrile (v/v)	0.2M Acetic acid
**Mobile phase C**	0.1% Formic acid in methanol (v/v)	Methanol
**Gradient linear**	40% B and 60% C	75% B and 25% C
**Retention time**	30 min	20 min
**UV detection**	360 nm	300 nm
**Temperature**	23 °C	30 °C
**Flow-rate**	1.2 mL/min	1 mL/min
